# Macrophage migration inhibitory factor–CD74 axis drives vascular smooth muscle cell–induced M1 macrophage polarization to exacerbate intracranial aneurysm inflammation

**DOI:** 10.3389/fimmu.2025.1682762

**Published:** 2025-11-26

**Authors:** Yao Chen, Jian-huang Huang, Qi-xiu Wang, Jian-hua Song, Jian-ning Chen

**Affiliations:** 1 Department of Neurosurgery, Affiliated Hospital of Putian University, Putian, Fujian, China; 2 Department of Cerebral Diseases Rehabilitation III, Affiliated Hospital of Liaoning University of Traditional Chinese Medicine, Shenyang, Liaoning, China; 3 Department of Clinical Medicine, Fujian Medical University, Fuzhou, Fujian, China

**Keywords:** macrophage migration inhibitory factor, CD74, secretory vascular smooth muscle cells, M1 macrophage polarization, intracranial aneurysms

## Abstract

**Background:**

Intracranial aneurysms (IAs) develop and progress through pathological processes, including inflammation and abnormal changes in the vascular structure. The cytokine Macrophage Migration Inhibitory Factor (MIF) is implicated in the pathology of vascular diseases. However, the role of MIF in IAs remains to be elucidated.

**Methods:**

Transcriptomic data from IA and normal arteries were analyzed to quantify MIF expression and immune infiltration (CIBERSORT). Methylation sequencing assessed MIF promoter methylation. Single-cell RNA sequencing (scRNA-seq) defined secretory vascular smooth muscle cell (sVSMC) and M1-like macrophage proportions and MIF expression. Intercellular communication via the MIF-CD74 axis was evaluated using CellChat. *In vitro* functional experiments validated sVSMC-induced macrophage M1 polarization mechanisms.

**Results:**

MIF mRNA was significantly upregulated in IAs (diagnostic AUC = 0.89) and correlated with increased M1-like macrophage infiltration (r = 0.783, p = 0.008). Hypomethylation of MIF was observed in IAs. scRNA-seq revealed expanded secretory VSMCs and M1-like macrophages, with elevated MIF in secretory VSMCs. CellChat confirmed enhanced MIF-CD74 signaling. *In vitro*, secretory VSMCs induced M1 polarization (iNOS/CD86↑, Arg1↓) via MIF-CD74; this effect was reversed by MIF knockdown or CD74 inhibition.

**Conclusion:**

We provide a comprehensive single-cell atlas of IAs and identify the sVSMC-derived MIF-CD74 axis as a novel mechanism driving macrophage M1 polarization and IA inflammation. This uncovers previously unrecognized sVSMC-macrophage crosstalk, establishing the MIF-CD74 axis as a promising immunomodulatory target for IA therapy.

## Introduction

Intracranial aneurysms (IAs) are a prevalent cerebrovascular disorder characterized by pathological dilation of major cerebral arteries, with an estimated prevalence of 3.2% (1). Unruptured intracranial aneurysms (UIAs) often remain asymptomatic for years. Increased utilization of cross-sectional imaging in clinical practice has heightened UIA detection rates. UIA rupture causes aneurysmal subarachnoid hemorrhage (aSAH), typically presenting with thunderclap headache or altered consciousness, and carries a poor prognosis: mortality exceeds 25% (2). Among survivors, one-third exhibit varying dependency on caregivers, while only one-fifth regain functional independence (3). Given the peak aSAH incidence occurs between 50–60 years of age, it results in a total loss of quality-adjusted life years (QALYs) comparable to ischemic stroke (4). Current management for high-risk UIAs involves preventive invasive interventions like surgical clipping or endovascular treatment, which carry significant risks of serious complications (5). Consequently, identifying novel non-invasive therapeutic strategies for UIAs is paramount.

Inflammation and aberrant vascular remodeling are pivotal pathological processes in IA development (6). Inflammation, particularly macrophage infiltration, critically contributes to IA formation and progression (7). Rodent IA models demonstrate substantial macrophage infiltration into the arterial wall during aneurysm formation (7), a phenomenon absent in normal cerebral arteries (8). Macrophage-driven chronic inflammation within the vascular wall is fundamental to IA progression (9). Notably, the dynamic interplay between pro-inflammatory M1-like and anti-inflammatory/reparative M2-like macrophage subtypes significantly influences IA initiation and progression (10). Early-stage IAs, characterized by mild structural alterations, are dominated by the M1-like phenotype (11, 12), which drives aneurysm formation and growth, leading to an increasing M1/M2 ratio over time (13). Conversely, M2-like macrophages are crucial for extracellular matrix clearance, vascular wall repair/remodeling, and inflammation resolution (14). Aberrant vascular remodeling is essential to IA pathogenesis. Under physiological conditions, vascular smooth muscle cells (VSMCs) synthesize collagen to counteract hemodynamic shear stress (8). However, during early aneurysm formation, inflammatory mediators concentrate within VSMCs (15), triggering their phenotypic modulation from a contractile to a secretory state. This transition features reduced expression of contractile markers (α-SMA, SM22α) and increased expression of matrix metalloproteinases (MMPs) (16). The precise relationship between secretory VSMCs and macrophages remains unclear and may represent a key mechanism underlying IA formation, progression, and rupture, warranting further investigation.

Macrophage migration inhibitory factor (MIF), an evolutionarily conserved, pleiotropic cytokine with upstream immunomodulatory functions (19), is a critical regulator in vascular pathologies (17, 18). Primarily secreted by monocytes, endothelial cells, and VSMCs (20), MIF expression is regulated by methylation and histone deacetylases (e.g., SIRT1) (21, 22). Following acute stress or inflammation, MIF is rapidly released and acts via autocrine/paracrine mechanisms (23, 24). MIF binding to receptors (CD74/CD44, CXCR4) activates downstream signaling pathways (ERK1/2, NF-κB, AKT), mediating pro-inflammatory responses implicated in aortic aneurysm, atherosclerosis, rheumatoid arthritis, and systemic lupus erythematosus (25–29). Notably, a Mendelian randomization study linked elevated serum MIF levels to UIAs (30). Recent work demonstrated that secretory VSMCs enhance immune cell function via MIF, recruiting immune cell infiltration into the arterial wall and promoting aortic aneurysm growth (29).

However, whether MIF mediates analogous pathological processes in IAs remains unexplored. Therefore, this study investigates whether secretory VSMCs induce M1 macrophage polarization via the MIF-CD74 axis in IAs. We will validate this mechanism using MIF knockdown and CD74 inhibition, providing novel insights into inflammatory regulation during IA pathogenesis.

## Materials and methods

### Bulk RNA sequencing data acquisition and processing

**Figure 1:**
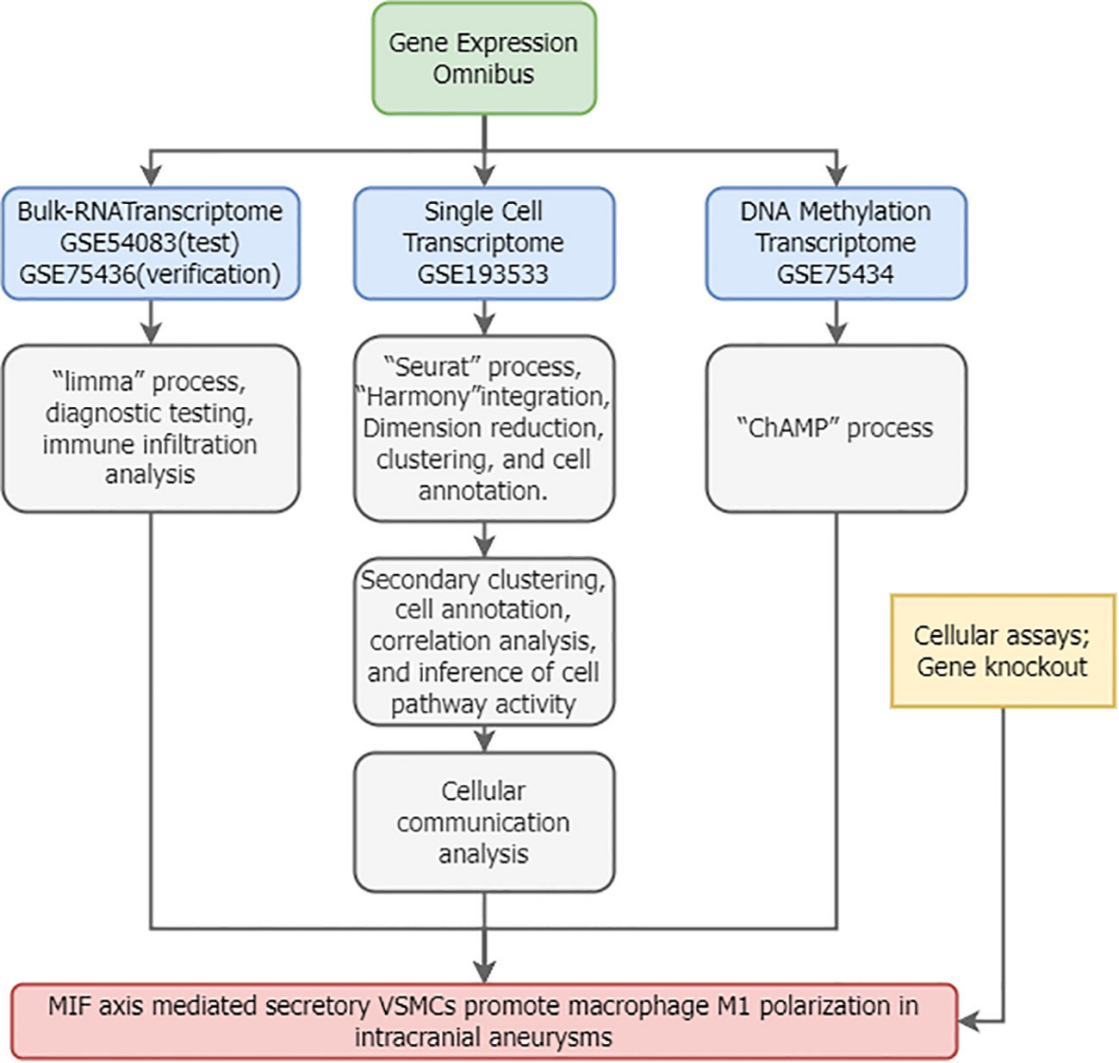
Study workflow.

**Figure 1** illustrates the overall study workflow. Bulk RNA sequencing (RNA-seq) transcriptomic datasets for intracranial aneurysms (IAs) were obtained from the Gene Expression Omnibus (GEO) database. The search strategy employed: (1) Topic: “intracranial aneurysm”; (2) Study type: “Expression profiling by array”; (3) Organism: *Homo sapiens*; (4) Inclusion of normal control samples. The gene-expression microarray dataset GSE54083 (platform GPL4133, Agilent-014850 Whole Human Genome Microarray 4x44K G4112F) comprised 8 ruptured IA samples, 5 UIA samples, and 10 superficial temporal artery (STA) control samples. Ruptured IA samples were excluded from this study. The gene-expression microarray dataset GSE75436 (platform GPL570, Affymetrix Human Genome U133 Plus 2.0 Array) contained 15 UIA samples and 15 STA controls. GSE54083 served as the test set and GSE75436 as the validation set.

### Differential expression analysis

Bulk RNA-seq data were processed using R (v4.2.1). During the raw data processing phase, we applied the RMA algorithm (for Affymetrix chips) and Agilent Feature Extraction software (for Agilent chips) based on the respective platform’s standard workflows. These were used to perform background correction, probe intensity normalization, and expression value summarization. Additionally, we utilized platform-specific probe annotation packages to map probes to genes. Data were normalized and log_2_-transformed with NormalizeBetweenArrays. Differentially expressed genes (DEGs) were identified using limma (31) (|log_2_FC| > 0.5, adj. p < 0.05).

### Single-cell data source and preprocessing

Single-cell RNA sequencing (scRNA-seq) data were obtained from the GEO dataset GSE193533. This dataset utilized single-cell suspensions derived from Willis circle arteries of adult male mice, including samples from sham-operated controls, elastase-induced unruptured IAs, and ruptured IAs. Our study incorporated one IA sample and one sham-treated arterial sample (32). Data integration and batch correction used Seurat (v4.3.2) and Harmony (v1.2.0) (33). Cells with <300 genes or >10% mitochondrial content were excluded. Count matrices were normalized (NormalizeData) and scaled (ScaleData).

### Cell type annotation

Top 2,000 highly variable genes (HVGs) were identified (FindVariableFeatures). Dimensionality reduction used principal component analysis (PCA) (top 20 PCs) and Uniform Manifold Approximation and Projection (UMAP) (resolution = 0.4). Clusters were manually annotated using canonical markers (32). Inter-cluster correlations were assessed via Spearman’s rank (p < 0.05).

### Cell-cell communication analysis

Cell-cell communication was inferred from scRNA-seq gene expression data using the CellChat package (v1.6.1). First, a global analysis incorporating all cell types identified differentially overexpressed ligands and receptors within each cell population, calculating statistically significant interactions (P < 0.05). Second, scRNA-seq data corresponding to M1-like macrophages and secretory vascular smooth muscle cells (VSMCs) were extracted to construct separate CellChat objects, enabling comparison of communication strength differences between groups. Significant interactions and communication strengths were visualized using circle plots and heatmaps.

### Gene enrichment analysis

DEGs underwent identifier conversion followed by enrichment analysis using the clusterProfiler package (v4.10) (34). Analyses included Gene Ontology Biological Process (GO_BP), Kyoto Encyclopedia of Genes and Genomes (KEGG) pathway enrichment (35, 36), and Gene Set Enrichment Analysis (GSEA) (37). Terms with a false discovery rate (FDR) < 0.05 were considered significantly enriched.

### Immune infiltration analysis

The CIBERSORT algorithm (38) was applied to the discovery cohort (GSE54083) to estimate immune cell type abundances in IAs versus controls. Pearson correlation evaluated associations between key gene expression and immune cell abundances (p<0.05).

### Pathway activity inference

Pathway activity was inferred from the fully annotated scRNA-seq data using the progeny package (v1.24) (39). PROGENy leverages a compendium of public perturbation experiments to generate pathway-specific gene signatures, enabling pathway activity inference from transcriptomic data.

### Methylation analysis

Methylation data for IAs were retrieved from GEO using the criteria: (1) Topic: “intracranial aneurysm”; (2) Study type: “Methylation profiling by array”; (3) Organism: *Homo sapiens*; (4) Inclusion of normal controls. The GSE75434 dataset, profiled on the GPL13534 platform (Illumina HumanMethylation450 BeadChip), included 9 unruptured IA samples and 9 STA control samples. Standard processing pipelines within the ChAMP R package (v2.32) (40) were used for data preprocessing, quality control (visualized via PCA and heatmaps using pheatmap), and differential methylation analysis. Differential methylation of MIF and CD74 loci was assessed using independent two-sample t-tests (*P* < 0.05), with results visualized via ggplot2.

### Cell culture and quality control

Human vascular smooth muscle cells (VSMCs, A7r5 cell line, ATCC CRL-1444) were cultured in DMEM (Gibco) supplemented with 10% fetal bovine serum (FBS, Gibco), 100 U/mL penicillin, and 100 µg/mL streptomycin (Sigma-Aldrich). THP-1 human monocytes (ATCC TIB-202) were differentiated into macrophages by treatment with 100 nM phorbol 12-myristate 13-acetate (PMA, Sigma-Aldrich) for 72 hours in RPMI-1640 medium (Gibco) containing 10% FBS. All cells were maintained at 37°C in a humidified 5% CO_2_ incubator. Cells between passages 3–5 were used for experiments. Cell morphology was routinely monitored using an inverted microscope (Olympus IX73). Mycoplasma contamination was assessed monthly using the MycoAlert™ Mycoplasma Detection Kit (Lonza); all cell batches tested negative. Cell viability, assessed by Trypan Blue (Sigma-Aldrich) exclusion, exceeded 95%. Culture medium was refreshed every 48 hours. All experimental procedures were conducted in a Class II biosafety cabinet. Environmental parameters (temperature, humidity, CO_2_ concentration) were monitored and recorded by the incubator system.

### Co-culture of secretory VSMCs and macrophages

To induce a secretory VSMC phenotype, VSMCs were stimulated with 10 ng/mL TNF-α (PeproTech) for 24 hours; controls received PBS. A Transwell co-culture system (0.4 µm pore, Corning) was established: upper chamber seeded with secretory or control VSMCs (1×10^5^ cells/well), lower chamber seeded with THP-1-derived macrophages (2×10^5^ cells/well). Experimental groups were: (1) Control VSMCs + Macrophages; (2) Secretory VSMCs + Macrophages; (3) Secretory VSMCs + Macrophages + 10 µg/mL anti-human MIF monoclonal antibody (Abcam); (4) Secretory VSMCs + Macrophages + 5 µM CD74 inhibitor BMS-582949 (MedChemExpress). After 48 hours of co-culture, macrophages from the lower chamber were collected for analysis.

### MIF knockdown experiment

VSMCs were transfected with MIF-specific siRNA (GenePharma, sequence: 5’-GCAUCAUGGUCUACGAUAA-3’) or negative control siRNA using Lipofectamine RNAiMAX (Invitrogen). Transfection efficiency was validated by qPCR 48 hours post-transfection. Cells were then stimulated with 10 ng/mL TNF-α for 24 hours to induce the secretory phenotype. The Transwell system was set up as above with MIF-knockdown or control VSMCs in the upper chamber and THP-1 macrophages in the lower chamber. Groups were: (1) Control VSMCs (siCtrl) + Macrophages; (2) Secretory VSMCs (siCtrl + TNF-α) + Macrophages; (3) MIF-knockdown Secretory VSMCs (siMIF + TNF-α) + Macrophages. Macrophages were collected after 48 hours.

### MIF-CD74 signaling inhibition assay

Conditioned medium (CM) was prepared by collecting serum-free medium from secretory VSMCs (stimulated with 10 ng/mL TNF-α for 24 hours), followed by filtration (0.22 µm, Millipore). THP-1 macrophages (2×10^5^ cells/well) were treated for 48 hours with: (1) Normal RPMI-1640 medium; (2) Secretory VSMC CM; (3) Secretory VSMC CM + 5 µM CD74 inhibitor BMS-582949. Macrophages were then collected.

### RNA extraction and quantitative PCR

Total RNA was isolated using TRIzol reagent (Invitrogen). Complementary DNA (cDNA) was synthesized using the PrimeScript RT reagent kit (Takara). qPCR was performed on a QuantStudio 5 system (Thermo Fisher Scientific) using SYBR Green Master Mix (Applied Biosystems). Primer sequences were: iNOS (F: 5’-CCTCGTCCCTGGCTTCCAA-3’, R: 5’-GCTGGGTCTCCTTCACTTCC-3’); Arg1 (F: 5’-GTGGAAACTTGCATGGACAAC-3’, R: 5’-AATCCTGGCACATCGGGAATC-3’); GAPDH (F: 5’-GGAGCGAGATCCCTCCAAAAT-3’, R: 5’-GGCTGTTGTCATACTTCTCATGG-3’). GAPDH served as the endogenous control. Relative gene expression was calculated using the 2^−ΔΔCt method. Each sample was analyzed in triplicate (n = 3 technical replicates per biological sample).

### Flow cytometry

Macrophages were stained with anti-CD86-PE antibody (1:200, BioLegend) for 30 minutes at 4°C. Following PBS washes, cells were analyzed on a CytoFLEX flow cytometer (Beckman Coulter). Data were processed using FlowJo software (v10.8) to determine the percentage of CD86^+^ cells. A minimum of 10,000 events per sample were acquired.

### Statistical analysis

All data processing and statistical analyses were performed using R software (version 4.3.3; R Core Team, 2023). Data wrangling, visualization, and analysis were conducted utilizing the following R packages: stats (v4.3.3), dplyr (v1.1.4), multcomp (v1.4-25), and ggplot2 (v3.5.1). Key findings were independently verified using GraphPad Prism 9.0 (GraphPad Software, Inc.). Data are presented as mean ± standard error of the mean (SEM). The qPCR experiment included 6 biological replicates per group, with each biological replicate subjected to 3 technical replicates.

Data normality was assessed using the Shapiro-Wilk test (stats::shapiro.test), and homogeneity of variances was evaluated using Levene’s test (car::leveneTest). For comparisons between groups meeting both normality and homoscedasticity assumptions, one-way analysis of variance (ANOVA; stats::aov) was applied, followed by Tukey’s honestly significant difference (HSD) *post hoc* test for multiple comparisons (multcomp::glht). When assumptions were violated, the non-parametric Kruskal-Wallis test (stats::kruskal.test) was employed, with Dunn’s *post hoc* test (FSA::dunnTest) for multiple comparisons. Statistical significance was defined as *P* < 0.05; denotes *P* < 0.01.

The reproducibility of qPCR and flow cytometry data was confirmed by a coefficient of variation (CV) < 10%. All statistical analyses were independently reviewed by two investigators. Raw R scripts and analytical outputs were archived to ensure full reproducibility and traceability.

## Results

### MIF as a diagnostic biomarker for IAs linked to M1 macrophage polarization

**Figure 2:**
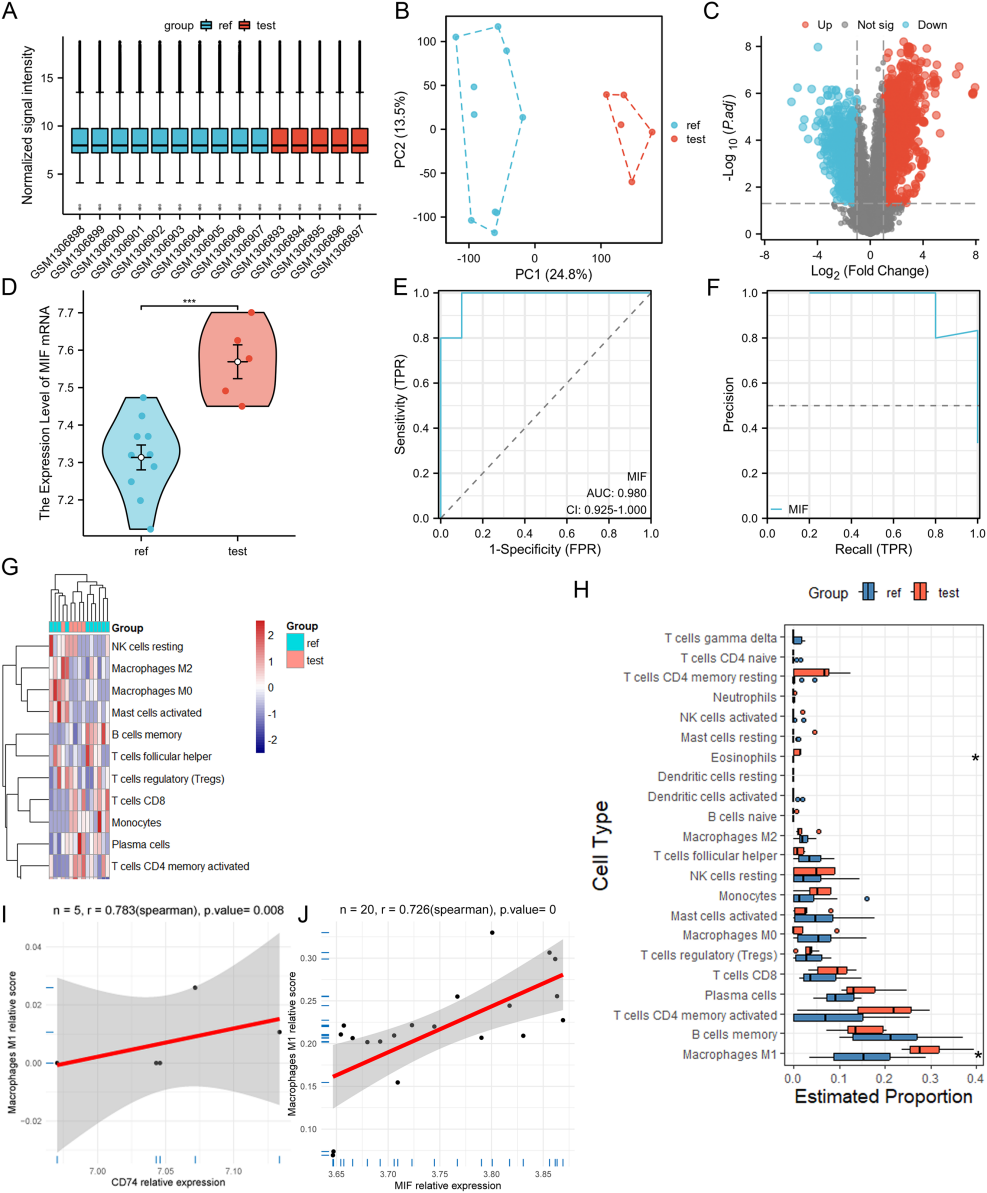
MIF is a robust diagnostic biomarker for IAs and correlates with macrophage M1 polarization. **(A-C)** Quality control metrics for dataset GSE54083. **(D)** Elevated MIF mRNA expression in IAs versus control arteries. **(E, F)** Diagnostic ROC and PR curves demonstrating the diagnostic utility of MIF for IAs. **(G, H)** Increased M1-like macrophage infiltration in IAs compared to controls. **(I, J)** Positive correlation between MIF mRNA expression and M1-like macrophage infiltration in the discovery (GSE54083) and validation (GSE75436) cohorts. ref: normal artery group; test: IA group; Up: differentially expressed genes with log_2_ fold change ≥1 and P < 0.05; Down: genes downregulated under the same criteria. *** and * indicate p-values less than 0.001 and 0.05, respectively.

High-quality gene-expression microarray data from GSE54083 showed clear separation between IAs and controls (**Figures 2A-C**). MIF expression was significantly elevated in IAs (**Figure 2D**). ROC analysis demonstrated excellent diagnostic performance (AUC = 0.980, CI:0.925-1.000), with PR curve precision >0.8 (**Figures 2E, F**). CIBERSORT revealed increased M1-like macrophage infiltration in IAs (**Figures 2G, H**), positively correlating with MIF expression (r=0.783, *P* = 0.008; **Figure 2I**). These findings were validated in GSE75436 (**Supplementary Figure S1**), maintaining strong correlation despite smaller initial sample size (N = 20, r=0.726, *P* < 0.0001; **Figure 2J**).

### Hypomethylation of MIF and CD74 in IAs

**Figure 3:**
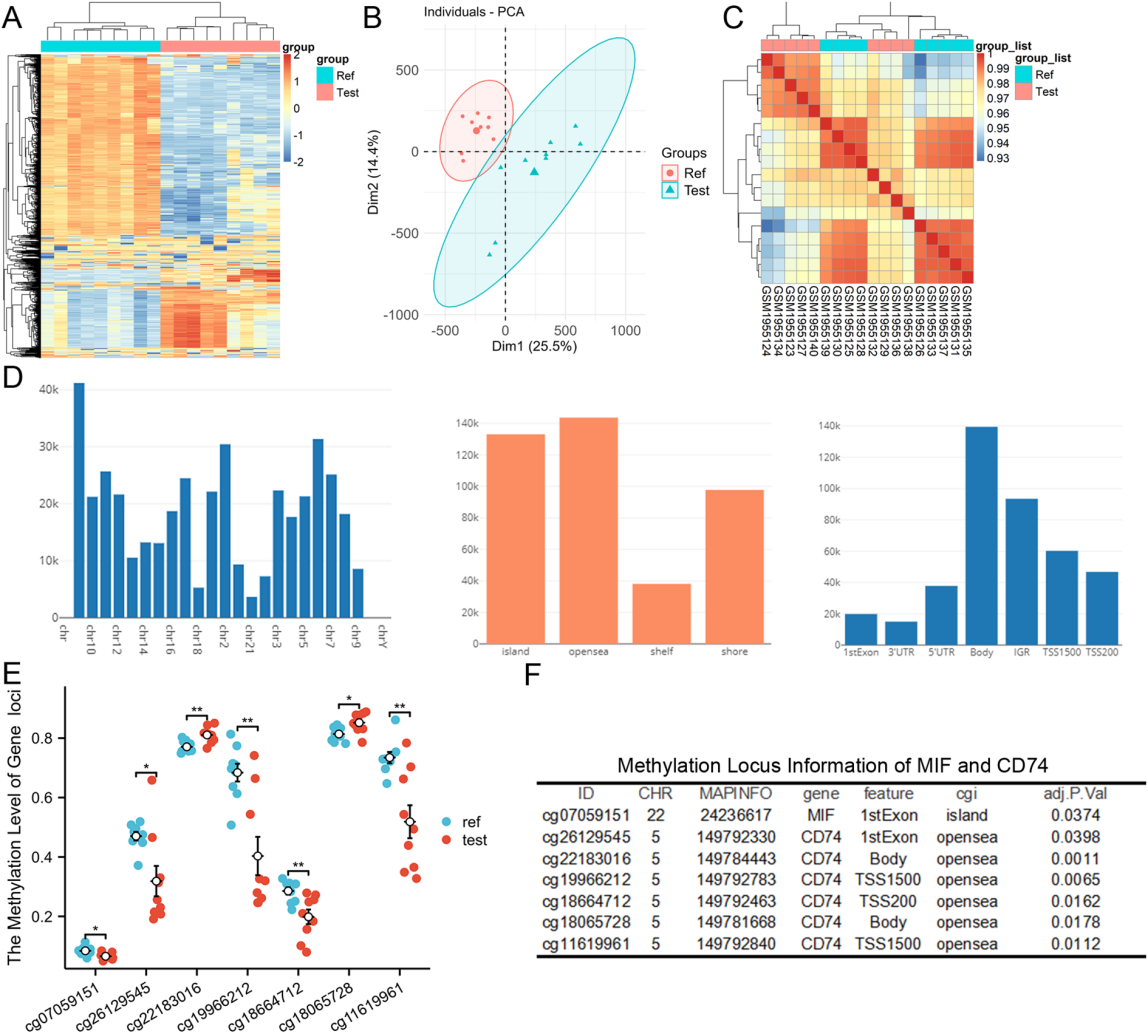
Hypomethylation of MIF and CD74 in dataset GSE75434. **(A-C)** Methylation data quality control. **(D)** Genomic distribution of methylation probes. **(E, F)** Significantly reduced methylation levels at MIF and CD74 loci in IAs versus control arteries. ** and * indicate p-values less than 0.01 and 0.05, respectively.

Quality control of the methylation dataset GSE75434 revealed no outliers. PCA demonstrated clear separation between IA and normal arterial samples, confirming distinct biological groups (**Figures 3A-C**). **Figure 3D** illustrates the genomic distribution of probes across the dataset. Differential methylation analysis identified significantly reduced methylation levels at specific loci within the MIF and CD74 genes in IAs compared to controls (**Figures 3E, F**). This hypomethylation likely contributes to the observed upregulation of MIF and CD74 mRNA expression in IAs.

### Single-cell Atlas construction and cell type annotation

**Figure 4:**
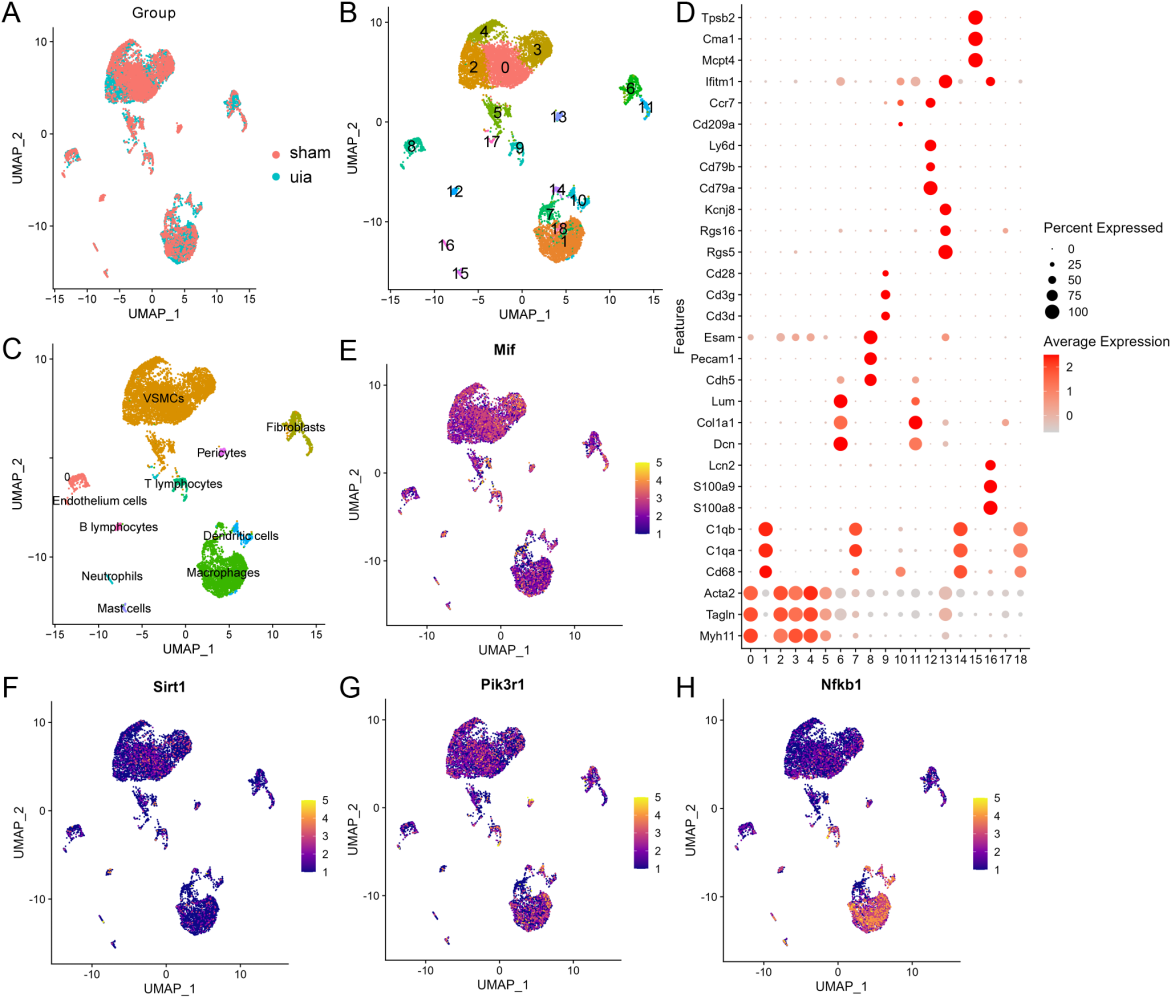
Single-cell transcriptomic atlas of dataset GSE193533. **(A)** Effective batch correction of IA and sham samples using Harmony. **(B)** UMAP visualization of 9,419 cells clustered into 19 distinct subpopulations. **(C)** Annotation of major cell lineages based on canonical markers. **(D)** Expression of lineage-defining marker genes across clusters. **(E-H)** Expression patterns of key MIF signaling pathway molecules (Mif, Pi3kr1, Sirt1, Nfkb1) across cell clusters.

Following stringent quality filtering (removing low-quality cells and genes), 9,419 cells and 17,821 genes were retained for analysis. Batch correction using Harmony effectively integrated samples (**Figure 4A**; detailed QC in **Supplementary Figure S2**). Unsupervised clustering (resolution = 0.4) partitioned these cells into 19 distinct clusters (**Figure 4B**). Manual annotation using established marker genes (32) categorized the cells into 10 major lineages: vascular smooth muscle cells (VSMCs), endothelial cells, fibroblasts, pericytes, and various immune cell populations (**Figure 4C**). Key marker genes for each cluster are visualized in **Figure 4D**.

Exploration of MIF pathway components revealed distinct expression patterns: Mif was highly expressed in VSMCs and macrophages, Pi3kr1 showed moderate expression in these cell types, Sirt1 was predominantly expressed in VSMCs followed by macrophages, and Nfkb1 was most abundant in macrophages (**Figures 4E-H**). This pattern suggests active MIF-mediated crosstalk between VSMCs and macrophages within IA tissue.

### Secretory VSMC phenotype predominates in IAs and exhibits functional alterations

**Figure 5:**
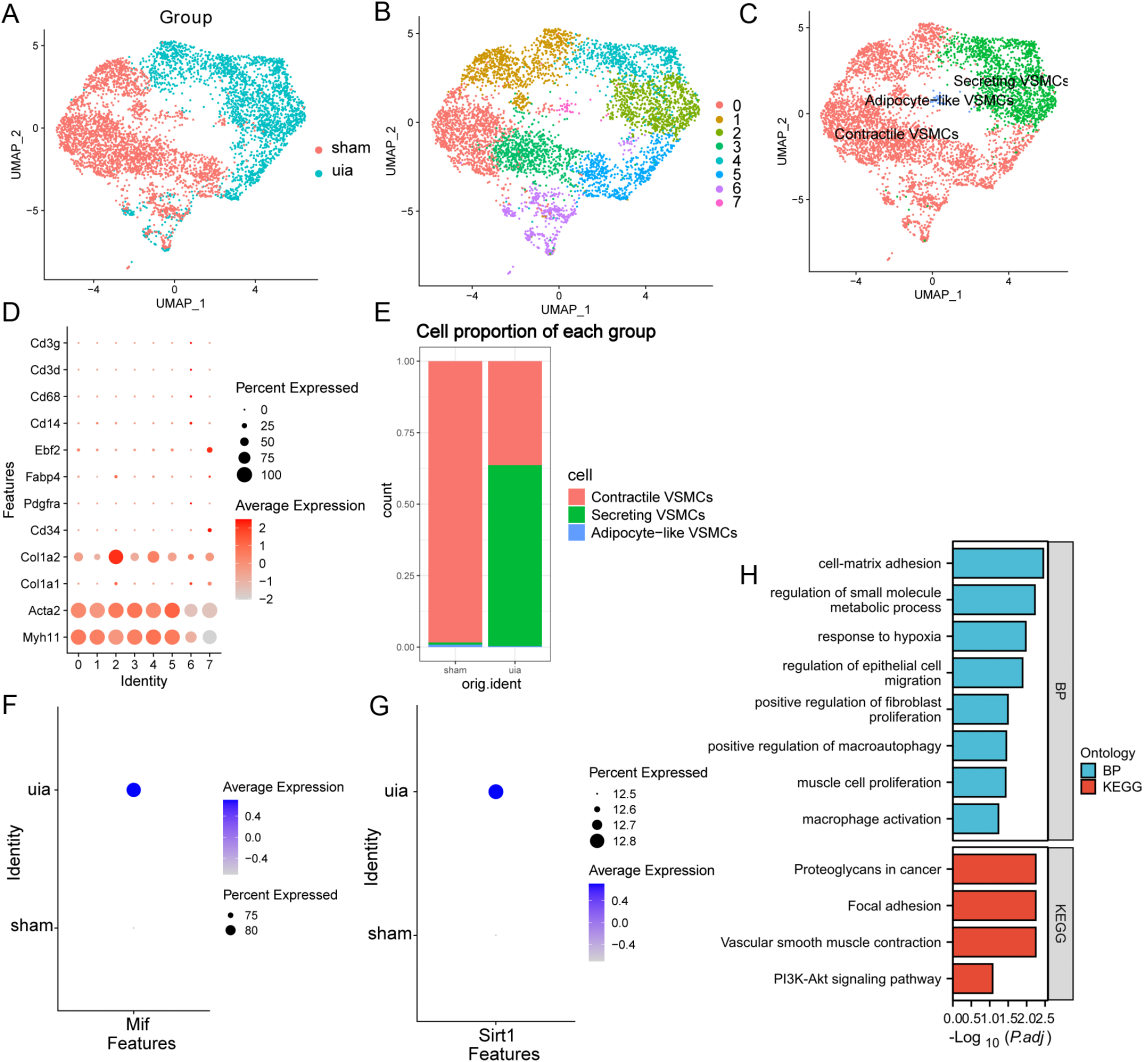
Single-cell characterization of vascular smooth muscle cells (VSMCs). **(A)** Effective batch correction of VSMC subsets. **(B)** UMAP visualization of 2,500 VSMCs clustered into 8 subpopulations. **(C)** Annotation of VSMC subtypes (secretory, contractile, other). **(D)** Expression of VSMC subtype markers across clusters. **(E)** Altered composition of VSMC subtypes in IAs versus sham controls. **(F, G)** Differential expression of Sirt1 and Mif in secretory VSMCs between groups. **(H)** GO and KEGG pathway enrichment analysis of DEGs in secretory VSMCs.

Subclustering of VSMCs after re-integration (**Figure 5A**) yielded 2,500 cells grouped into 8 clusters (**Figure 5B**). Annotation using VSMC markers identified a predominance of secretory VSMCs (characterized by ACTA2^+^, MYH11^+^, COL1A1^+^, COL1A2^+^), followed by contractile VSMCs, with other phenotypes being sparse (**Figures 5C, D**). The proportion of secretory VSMCs was significantly elevated in IAs, concomitant with a reduction in contractile VSMCs (**Figure 5E**). Importantly, secretory VSMCs in IAs exhibited altered expression levels of Sirt1 and Mif compared to controls (**Figures 5F, G**).

Differential gene expression analysis of secretory VSMCs identified upregulated genes (e.g., Tns1, Itga9) and downregulated genes (e.g., Anxa1, S100a10) in IAs (**Supplementary Figure S3A**). Gene Ontology (GO) and KEGG pathway enrichment analyses of DEGs implicated processes including macrophage activation, positive regulation of macroautophagy, positive regulation of vascular smooth muscle cell migration, and PI3K-Akt signaling pathway (**Figure 5H**). These findings highlight the potential role of secretory VSMCs in IA pathogenesis, particularly through interactions with macrophages.

### Macrophage identification and functional analysis

**Figure 6:**
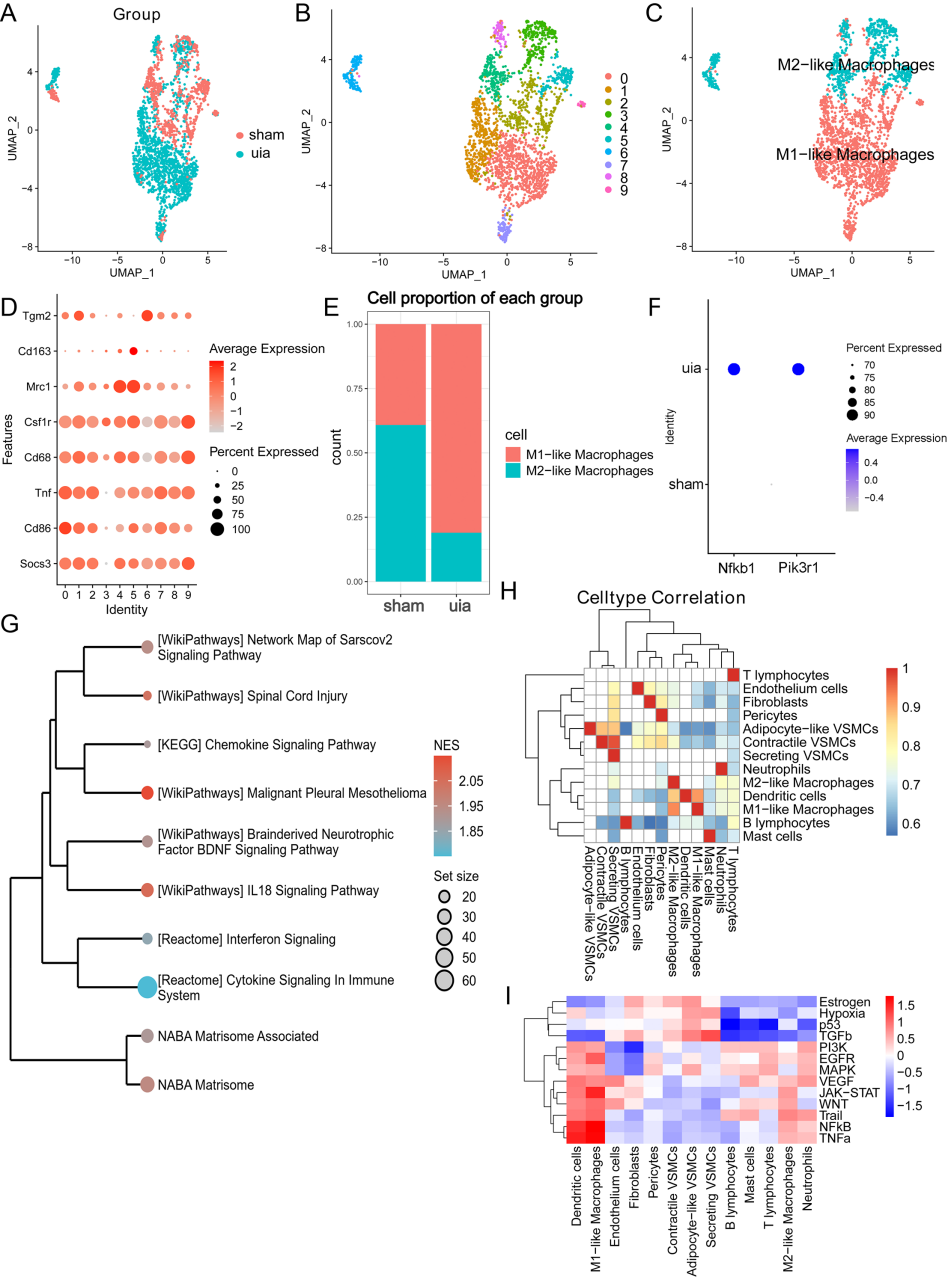
Characterization and functional analysis of macrophages. **(A)** Effective batch correction of macrophage subsets. **(B)** UMAP visualization of 2,414 macrophages clustered into 10 subpopulations. **(C)** Annotation of M1-like and M2-like macrophage subsets. **(D)** Expression of macrophage subset markers across clusters. **(E)** Increased proportion of M1-like macrophages in IAs. **(F)** Elevated expression of Pik3r1 and Nfkb1 in M1-like macrophages from IAs. **(G)** GSEA enrichment plot highlighting pro-inflammatory pathways in M1-like macrophages. **(H)** Spearman correlation heatmap showing interrelationships between cell type abundances. **(I)** Heatmap depicting inferred pathway activity scores across major cell types.

Subclustering of macrophages after re-integration (**Figure 6A**) yielded 2,414 cells grouped into 10 clusters (**Figure 6B**). Although the novel binary classification system (TREM2+SPP1+ and FOLR2+ macrophages) failed the marker exclusivity test (**Supplementary Figures S4**-**6**), the core markers (CD86/MRC1) of the classical M1/M2 classification demonstrated stable mutual exclusivity in high-dimensional data (**Figure 6C**). Given the pathology-dependent nature of macrophage phenotypes, this study prioritizes the application of the classical classification framework. Marker gene expression across clusters is shown in **Figure 6D**.

The proportion of M1-like macrophages was significantly increased in IAs (**Figure 6E**), and these cells exhibited elevated expression of Pik3r1 and Nfkb1 compared to controls (**Figure 6F**). Differential gene expression analysis of M1-like macrophages identified upregulated genes (e.g., Oasl1, Cxcl10) and downregulated genes (e.g., Snrpf, Pim3) in IAs (**Supplementary Figure S3B**). Gene Set Enrichment Analysis (GSEA) of all genes confirmed a strong enrichment for pro-inflammatory pathways in M1-like macrophages from IAs (**Figure 6G**), underscoring their critical role in IA pathogenesis.

Spearman correlation analysis revealed a significant positive association between the abundance of secretory VSMCs and M1-like macrophages (r = 0.68, P < 0.05; **Figure 6H**). Inference of pathway activity using PROGENy indicated prominent activation of TGFβ, p53, and hypoxia pathways in secretory VSMCs, while M1-like macrophages showed upregulation of NFκB and TNFα signaling (**Figure 6I**). This suggests functional interplay between secretory VSMCs and M1-like macrophages contributes to IA development and progression.

### Enhanced MIF-CD74/CD44 axis signaling mediates secretory VSMC - M1-like macrophage crosstalk in IAs

**Figure 7:**
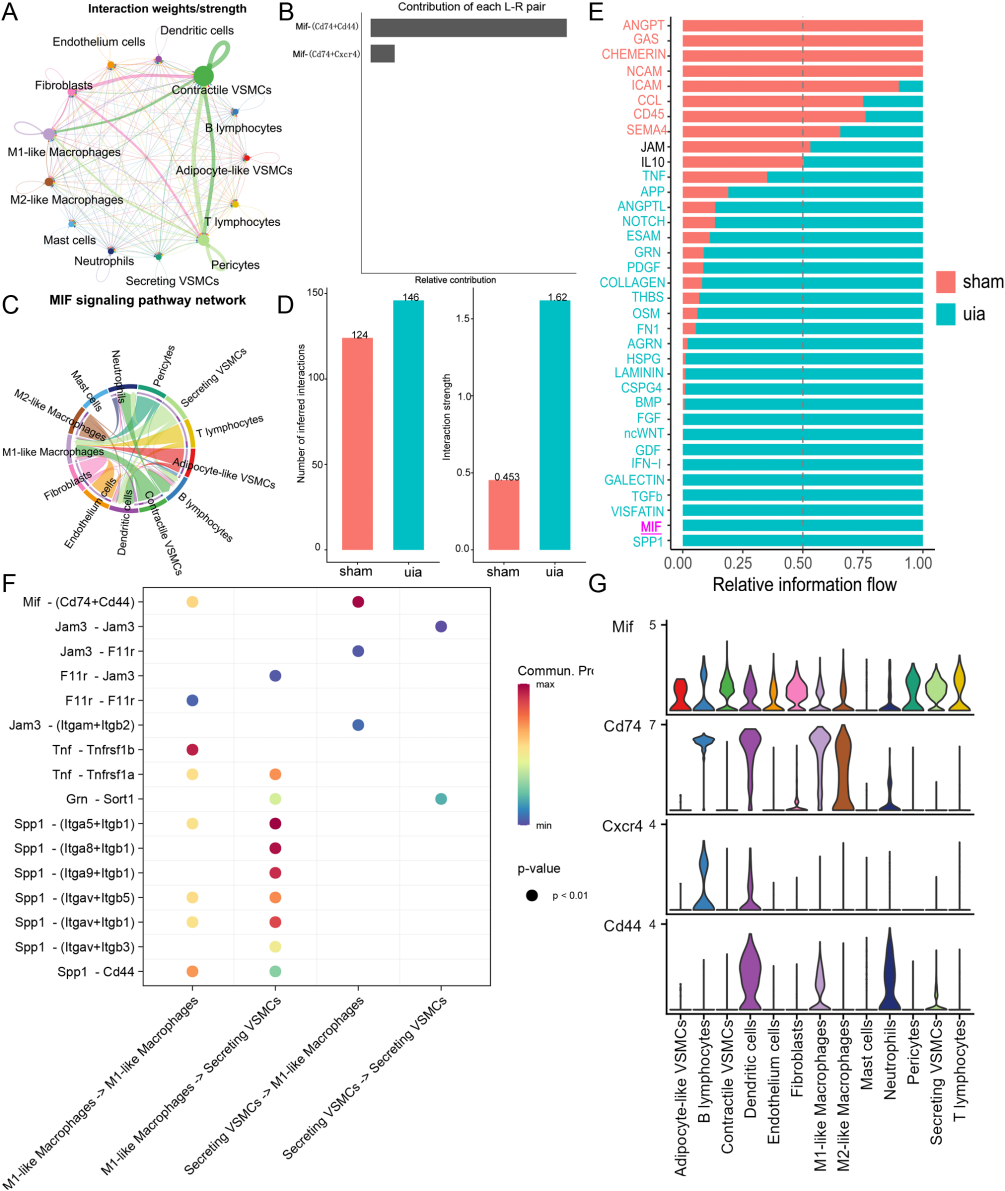
Cell-cell communication analysis. **(A)** Global communication network showing interaction strength between major cell types. **(B)** Interaction weights for MIF ligand-receptor pairs. **(C)** Chord diagram highlighting MIF-CD74/CD44 signaling between VSMCs and M1-like macrophages. **(D)** Increased interaction strength and number between secretory VSMCs and M1-like macrophages in IAs. **(E)** Differential signaling pathway activity between secretory VSMCs and M1-like macrophages across conditions. **(F)** Heatmap showing communication probability for key pathways between secretory VSMCs and M1-like macrophages. **(G)** Expression patterns of key MIF axis components (Mif, Cd74, Cd44) across cell types.

Global cell-cell communication analysis revealed extensive interactions across cell types, particularly between VSMCs, fibroblasts, and macrophages (**Figure 7A**). Investigation of MIF signaling identified MIF-CD74 and MIF-CD44 as the predominant ligand-receptor pairs (**Figure 7B**). Chord diagram visualization confirmed the MIF axis as a major communication pathway between VSMCs and M1-like macrophages (**Figure 7C**).

Focused analysis on secretory VSMCs and M1-like macrophages demonstrated a significant increase in both the number and strength of interactions in IAs compared to controls, where interactions were minimal (**Figure 7D**). Identification of conserved and context-specific signaling pathways further revealed significantly enhanced MIF axis signaling between secretory VSMCs and M1-like macrophages in IAs (**Figure 7E**). Expression analysis confirmed moderate MIF-CD74/CD44 signaling potential between these cell types (**Figures 7F, G**).

### Secretory VSMCs promote macrophage M1 polarization via the MIF-CD74 axis

**Figure 8:**
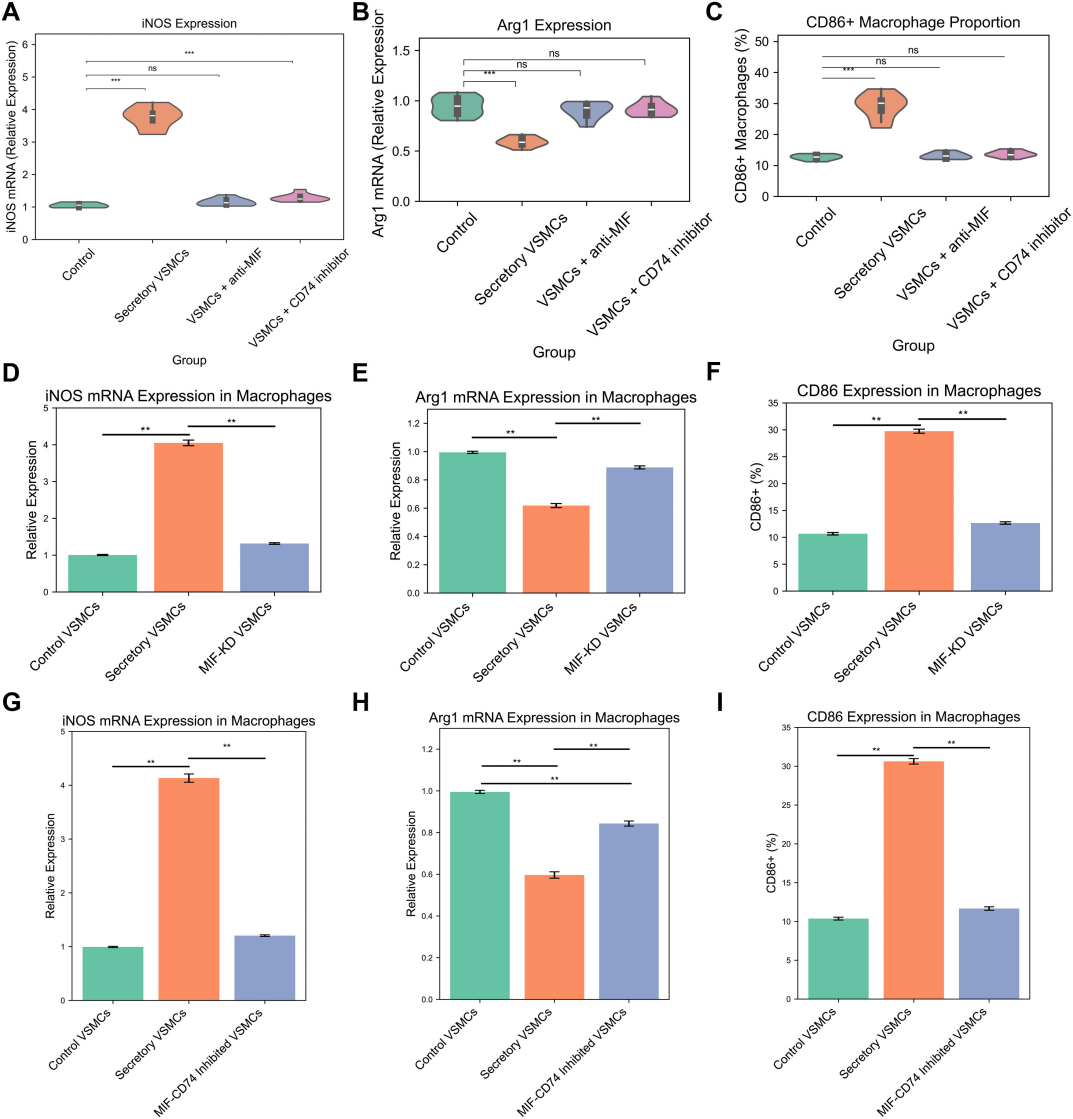
Secretory VSMCs drive macrophage M1 polarization via the MIF-CD74 axis. **(A, B)** qPCR analysis of iNOS and Arg1 expression in macrophages following Transwell co-culture. Secretory VSMCs increase iNOS and decrease Arg1; anti-MIF antibody or CD74 inhibitor (BMS-582949) attenuates this effect. **(C)** Flow cytometry quantification of CD86^+^ macrophages. Secretory VSMCs increase CD86^+^ proportion; inhibition of MIF or CD74 reduces it. **(D, E)** qPCR validation of MIF knockdown efficiency in VSMCs and its effect on macrophage iNOS/Arg1 expression in co-culture. MIF knockdown reverses secretory VSMC-induced polarization. **(F)** Flow cytometry shows MIF knockdown reduces CD86^+^ macrophage proportion induced by secretory VSMCs. **(G, H)** qPCR analysis of macrophages treated with secretory VSMC conditioned medium (CM). CM increases iNOS and decreases Arg1; CD74 inhibition reverses this. **(I)** Flow cytometry shows CD74 inhibition reduces CM-induced increase in CD86^+^ macrophages. Data are mean ± SEM; P < 0.01, P < 0.05 versus control; ##P < 0.01, #P < 0.05 versus secretory VSMC group (One-way ANOVA with Tukey’s *post hoc* test).

Transwell co-culture experiments functionally validated the impact of secretory VSMCs on macrophage polarization. qPCR analysis revealed that co-culture with secretory VSMCs significantly upregulated iNOS mRNA expression (3.52 ± 0.20 vs. 1.00 ± 0.05; P < 0.01) and downregulated Arg1 mRNA expression (0.65 ± 0.04 vs. 1.00 ± 0.05; P < 0.01) in macrophages compared to controls (**Figures 8A, B**). This effect was significantly attenuated by either an anti-MIF blocking antibody (iNOS: 1.18 ± 0.07, P < 0.01; Arg1: 0.91 ± 0.05, P < 0.05) or the CD74 inhibitor BMS-582949 (iNOS: 1.22 ± 0.08, P < 0.01; Arg1: 0.89 ± 0.05, P < 0.05). Flow cytometry confirmed a substantial increase in the percentage of CD86^+^ M1-like macrophages following co-culture with secretory VSMCs (29.1% ± 1.8% vs. 5.6% ± 0.5%; P < 0.01), which was markedly reduced by anti-MIF antibody (11.5% ± 0.7%; P < 0.01) or BMS-582949 (12.3% ± 0.8%; P < 0.01) treatment (**Figure 8C**). The corresponding gating strategy is shown in **Supplementary Figure S7A**.These results demonstrate that secretory VSMCs drive macrophage M1 polarization predominantly through the MIF-CD74 axis.

### MIF knockdown attenuates secretory VSMC-induced M1 polarization

Specific MIF knockdown in VSMCs (achieving 84.8% ± 1.1% reduction in mRNA; P < 0.01; **Figures 8D, E**) significantly mitigated their pro-M1 polarizing effect. Co-culture with control secretory VSMCs robustly increased macrophage iNOS (3.98 ± 0.22 vs. 1.00 ± 0.05; P < 0.01) and decreased Arg1 (0.62 ± 0.04 vs. 1.00 ± 0.05; P < 0.01). MIF knockdown abolished these changes (iNOS: 1.25 ± 0.08, P < 0.01; Arg1: 0.88 ± 0.05, P < 0.05). Flow cytometry similarly showed that MIF knockdown prevented the secretory VSMC-induced increase in CD86^+^ macrophages (12.0% ± 0.8% vs. 30.5% ± 1.9% in control secretory co-culture; P < 0.01; **Figure 8F**), confirming the essential role of VSMC-derived MIF. The corresponding gated graph is shown in **Supplementary Figure S7B**.

### CD74 inhibition reverses MIF-mediated M1 polarization

Conditioned medium (CM) from secretory VSMCs recapitulated the M1-polarizing effect, significantly increasing macrophage iNOS (3.75 ± 0.21 vs. 1.00 ± 0.05; P < 0.01) and decreasing Arg1 (0.64 ± 0.04 vs. 1.00 ± 0.05; P < 0.01) expression (**Figures 8G, H**). Treatment with the CD74 inhibitor BMS-582949 effectively reversed these effects (iNOS: 1.15 ± 0.06, P < 0.01; Arg1: 0.92 ± 0.05, P < 0.05). Flow cytometry confirmed that CM increased CD86^+^ macrophages (28.3% ± 1.7% vs. 5.5% ± 0.5%; P < 0.01), and this increase was significantly blunted by CD74 inhibition (11.3% ± 0.6%; P < 0.01; **Figure 8I**), demonstrating that blocking CD74 signaling interrupts MIF-mediated M1 polarization. The corresponding gated graph is shown in **Supplementary Figure 7C**.

## Discussion

This study presents a comprehensive single-cell atlas of intracranial aneurysms (IAs) and reveals that secretory vascular smooth muscle cells (VSMCs) induce macrophage M1 polarization via the MIF-CD74 axis. Secretory VSMCs significantly upregulated macrophage iNOS mRNA and CD86+ cell proportion while downregulating Arg1 mRNA, indicating M1 polarization. These effects were reversed by anti-MIF antibody, MIF knockdown, or CD74 inhibition, confirming the critical role of the MIF-CD74 axis. These findings align with reports that MIF promotes pro-inflammatory macrophage responses via CD74-mediated NF-κB activation (29). Crucially, we demonstrate that secretory VSMCs act as key microenvironmental regulators, driving M1 polarization via MIF secretion and CD74 activation, potentially amplifying inflammatory cascades in atherosclerosis. This provides a foundation for understanding IA pathogenesis. Notably, this work may be the first to report the contribution of the MIF-CD74 axis to unruptured IAs (UIAs).

MIF is critically involved in UIA pathology. We observed significant upregulation of MIF mRNA in UIAs, consistent with reports in aortic aneurysms (29, 41). MIF is constitutively expressed and stored in most mammalian cells, playing essential roles in various physiological processes. Unlike typical cytokines, MIF release is triggered by pro-inflammatory stimuli or cellular stress, followed by replenishment via transcriptional upregulation and translation (23). Our data suggest potential mechanisms for MIF overexpression in UIAs: hypomethylation of the MIF gene locus and elevated expression of the histone deacetylase Sirt1. Supporting this, both bulk RNA-seq and scRNA-seq revealed markedly increased MIF mRNA levels in IA walls compared to normal arteries, reinforcing the presence of sustained inflammation (29, 42). However, this study has not directly measured the protein expression level of MIF in IA tissues. In the future, we will further validate the expression changes of MIF protein in IA through experiments such as immunohistochemistry, Western blot, or ELISA to strengthen its reliability as a diagnostic marker and therapeutic target. The high diagnostic accuracy of MIF (demonstrated by ROC and PR curves) further underscores its close involvement in UIA pathophysiology. Crucially, immune deconvolution analysis revealed not only increased M1-like macrophage abundance in UIAs but also a significant positive correlation between MIF expression and M1-like macrophage infiltration, strongly suggesting MIF mediates macrophage recruitment and/or polarization.

Single-cell transcriptomics further delineated the source and mode of action of MIF in UIAs. We confirmed high MIF expression in both VSMCs and macrophages (29), with secretory VSMCs—significantly expanded in UIAs—representing a major source. In contrast, MIF receptor expression was heterogeneous. Combinations like CD74/CD44 or CD74/CXCR4 were predominantly expressed on immune cells, with CD74 highly abundant on M1-like macrophages. Downstream effectors PI3K (Pik3r1) and NFκB (Nfkb1) were also highly expressed in UIA macrophages, aligning with their increased M1-like polarization (43, 44). Spearman correlation analysis further revealed a significant positive association between secretory VSMC and M1-like macrophage abundances. We therefore propose the MIF axis as a key mechanism by which secretory VSMCs drive M1 macrophage polarization within the UIA microenvironment. It is noteworthy that although alternative classification schemes (such as TREM2+SPP1+ and FOLR2+) may reflect the functional heterogeneity of macrophages, quantitative analysis reveals partial overlap with classical M1/M2 markers. In this study, M1 and M2 cells remain the most significantly proportionally distinct and functionally opposing subpopulations in IA tissues. Therefore, the M1/M2 classification is retained to focus on the core mechanisms of inflammatory polarization.

Cell-cell communication analysis substantiated this hypothesis. Global analysis revealed active MIF signaling between VSMCs and M1-like macrophages. Notably, while MIF is basally expressed in normal arteries, significant MIF-mediated interactions between secretory VSMCs and M1-like macrophages were specifically enhanced in UIAs. CD74/CD44 was the dominant receptor complex for MIF on M1-like macrophages. CD44 typically functions as a co-receptor for CD74, enhancing the endocytosis and signal transduction of the MIF-CD74 complex. However, its individual role is relatively weak, and it primarily assists CD74 in exerting its effects. In our data, CD74 is predominantly expressed in M1 macrophages and is the receptor most significantly associated with MIF signaling intensity. Therefore, we prioritize focusing on the functional validation of CD74. Although CXCR4 is also one of the receptors for MIF, its expression in macrophages is relatively low, and its primary functions are more related to processes such as cell migration and chemotaxis, rather than directly regulating M1/M2 polarization. Through CellChat and pathway activity analysis, we identified the MIF-CD74/CD44 pair as the most prominent ligand-receptor interaction between VSMCs and M1 macrophages, thus selecting it as the primary subject for further investigation. Unlike bidirectional signaling often observed, our analysis specifically highlighted the MIF axis as a crucial pathway from secretory VSMCs to M1-like macrophages, as no other pathway exhibited comparable strength or specificity in this direction. Importantly, CD44 and CXCR4 engagement can activate NFκB signaling (45, 46), which is fundamental for establishing and maintaining the M1 phenotype.

Functional validation confirmed that the pro-inflammatory effect of secretory VSMCs on macrophages is mediated by soluble MIF, not direct cell contact, as demonstrated by Transwell co-culture and conditioned medium experiments. Specific MIF knockdown significantly attenuated M1 polarization (iNOS↑, Arg1↓, CD86^+^%), confirming MIF as the primary mediator. Furthermore, CD74 inhibition effectively reversed M1 polarization, identifying the MIF-CD74 axis as a promising therapeutic target. These results corroborate the work of Cao et al. (29, 41) while directly establishing, through *in vitro* models, the mechanistic role of VSMC-macrophage crosstalk via MIF-CD74, complementing limitations inherent to *in vivo* studies. Although this study did not directly measure the phosphorylation status of NFκB, PI3K, or MAPK pathways, their potential involvement is supported by the following evidence (47–49): (1) The canonical downstream signaling of the MIF-CD74 axis includes NFκB (regulating pro-inflammatory genes such as iNOS) and PI3K/AKT (maintaining the inflammatory phenotype of macrophages); (2) The functional phenotypes observed in this study (upregulation of iNOS, increased proportion of CD86+ cells) are highly consistent with the known effects of these pathways; (3) Future experiments will utilize Western blot to measure the levels of p-p65, p-AKT, and p-ERK in macrophages treated with secretory VSMC-conditioned medium, in order to validate the inhibitory effect of CD74 blockade on these phosphorylation events and thereby refine the mechanistic cascade.

In summary, we identified the MIF axis as a critical pathway by which secretory VSMCs promote M1-like macrophage polarization in UIAs and validated MIF-CD74 as a potential therapeutic target. This provides a fundamental advance in understanding IA pathogenesis and offers a novel rationale for immunomodulatory strategies. Our study has limitations. Primarily, the *in vitro* models, while informative, cannot fully recapitulate the complexity of the *in vivo* microenvironment, including contributions from other immune cells or cytokines. Secondly, downstream signaling cascades of the MIF-CD74 axis (e.g., NF-κB, MAPK) were not mechanistically dissected; future studies should employ phospho-proteomics or specific pathway inhibitors. It is noteworthy that the single-cell RNA sequencing data used in this study were derived from a mouse UIA model, while the DNA methylation analysis data came from human samples, indicating certain interspecies differences between the two. Although mice and humans share high similarities in the pathological features of UIA—such as inflammatory cell infiltration and phenotypic switching of vascular smooth muscle cells—and MIF, as a highly conserved cytokine, exhibits well-documented conservation in its signaling mechanisms (e.g., binding to CD74/CD44, activation of NF-κB) across species including humans, mice, and rats, which supports the potential functional similarity of the MIF-CD74 axis in regulating macrophage polarization between humans and mice, the molecular regulatory networks across species may still differ. Therefore, we interpret the cell subpopulations identified at the single-cell level and their interaction patterns with caution, and integrate human bulk transcriptome and methylation data for comprehensive analysis to collectively support the key role of MIF in UIA. Based on the above findings and limitations, future studies should further validate the functional significance of the MIF signaling axis in more clinically relevant model systems. We plan to establish stable and reliable animal models, conduct loss-of-function experiments (such as gene knockout or neutralization antibody interventions) and targeted inhibitor treatment experiments to further investigate the *in vivo* function of the MIF-CD74 axis (including its effects on M1 polarization, the inflammatory microenvironment, and aneurysm progression). These experiments will help systematically validate the molecular hypotheses proposed in this study and further refine the relevant theoretical framework. Admittedly, such functional experiments typically require substantial time and research resources. Nevertheless, their value in advancing the translation of UIA immunotherapeutic targets is significant, warranting in-depth exploration and continued efforts.

## Conclusion

This study constructs a comprehensive single-cell atlas of IAs and defines a pivotal role for the MIF-CD74 axis in driving pathogenic inflammation via secretory VSMC-mediated M1 macrophage polarization. Targeting MIF or CD74 represents a promising immunomodulatory strategy for IA management, meriting further preclinical and clinical exploration.

## Data Availability

The datasets analyzed in this study are available for download in the Gene Expression Omnibus (GEO). GSE54083 dataset: https://www.ncbi.nlm.nih.gov/geo/query/acc.cgi?acc=gse54083, GSE75436dataset: https://www.ncbi.nlm.nih.gov/geo/query/acc.cgi?acc=gse75436, GSE193533 dataset: https://www.ncbi.nlm.nih.gov/geo/query/acc.cgi?acc=gse193533, GSE75434 dataset: https://www.ncbi.nlm.nih.gov/geo/query/acc.cgi?acc=GSE75434.
