# Supplementary material for: Macrophage migration inhibitory factor–CD74 axis drives vascular smooth muscle cell–induced M1 macrophage polarization to exacerbate intracranial aneurysm inflammation
Source: Front Immunol. 2025 Nov 26;16:1682762. doi: 10.3389/fimmu.2025.1682762 (PMC12689327; doi:10.3389/fimmu.2025.1682762)
Supplement: Supplementary file 1 [file DataSheet1.doc]

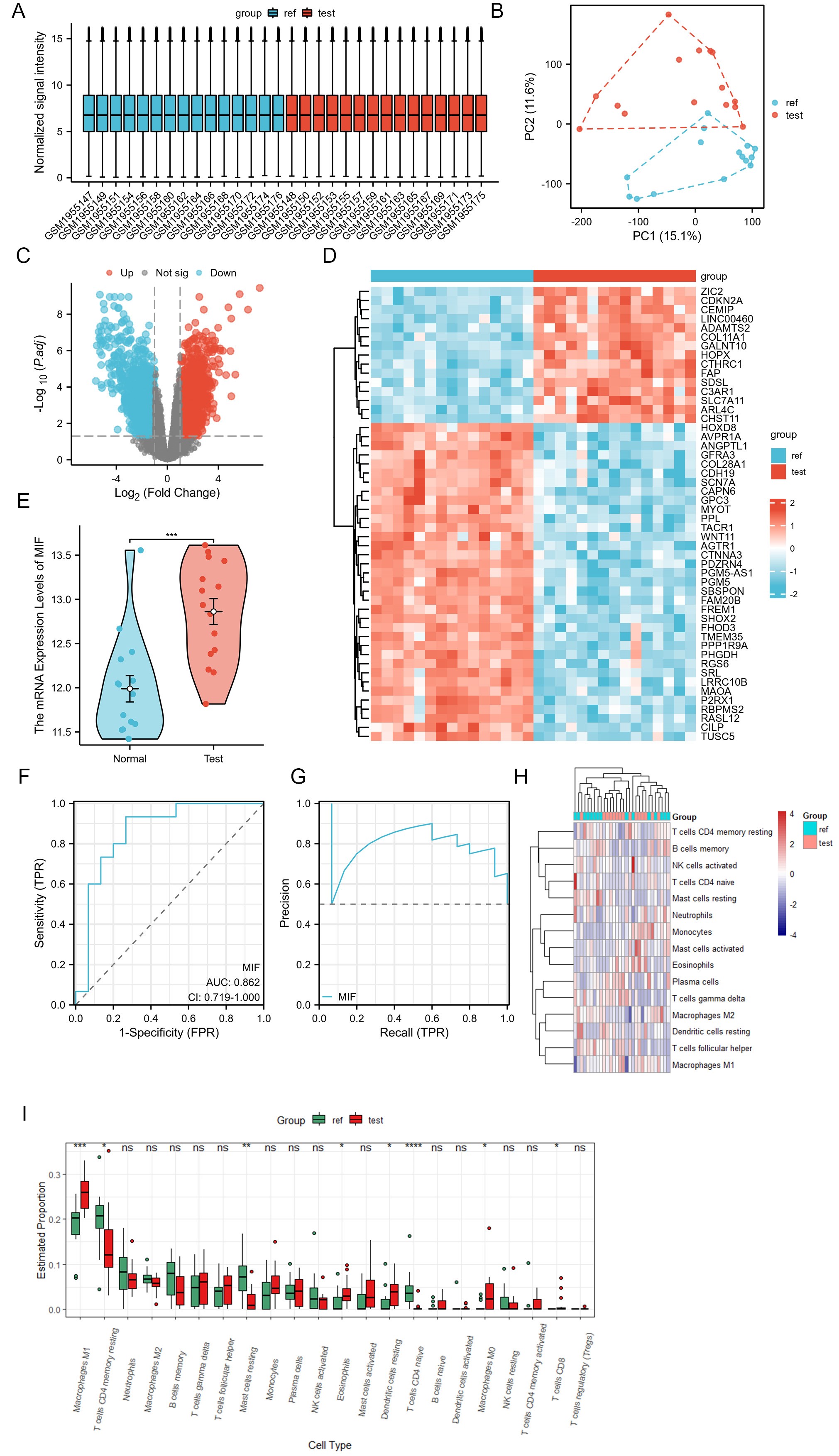


Figure S1. **MIF is a good diagnostic indicator for IAs and may be related to M1 polarization of macrophages**. A-D. The data quality control of dataset GSE75436 is qualified. E. The expression level of MIF mRNA in IAs is higher than that in control samples. F-G. The ROC curve and PR curve for MIF diagnosis suggest that MIF has high diagnostic value for IAs. H-I. The infiltration level of M1-like macrophages in IAs is higher than that in control samples.


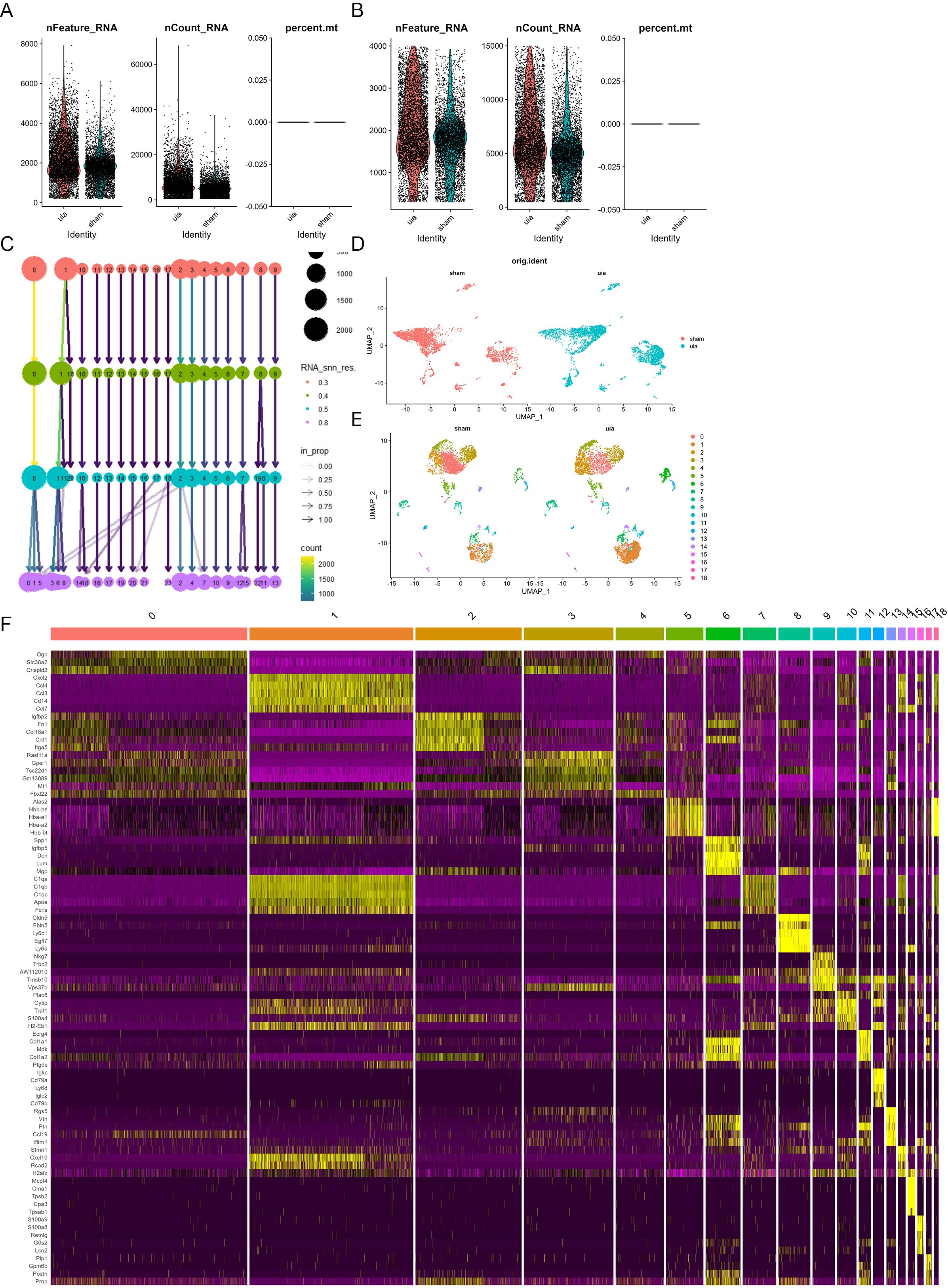


Figure S2. **Quality control and general process of single-cell data.** A. Data distribution before quality control of single-cell data. B. Data distribution after quality control of single-cell data. C. Clustering of single-cell data at different resolutions using the "clustree" package. D-E. UMAP distribution of two groups of data before and after integration using the harmony method for single-cell data. F. Heatmap showing the clustering of single-cell data at a resolution of 0.4 and the identification of highly expressed genes.


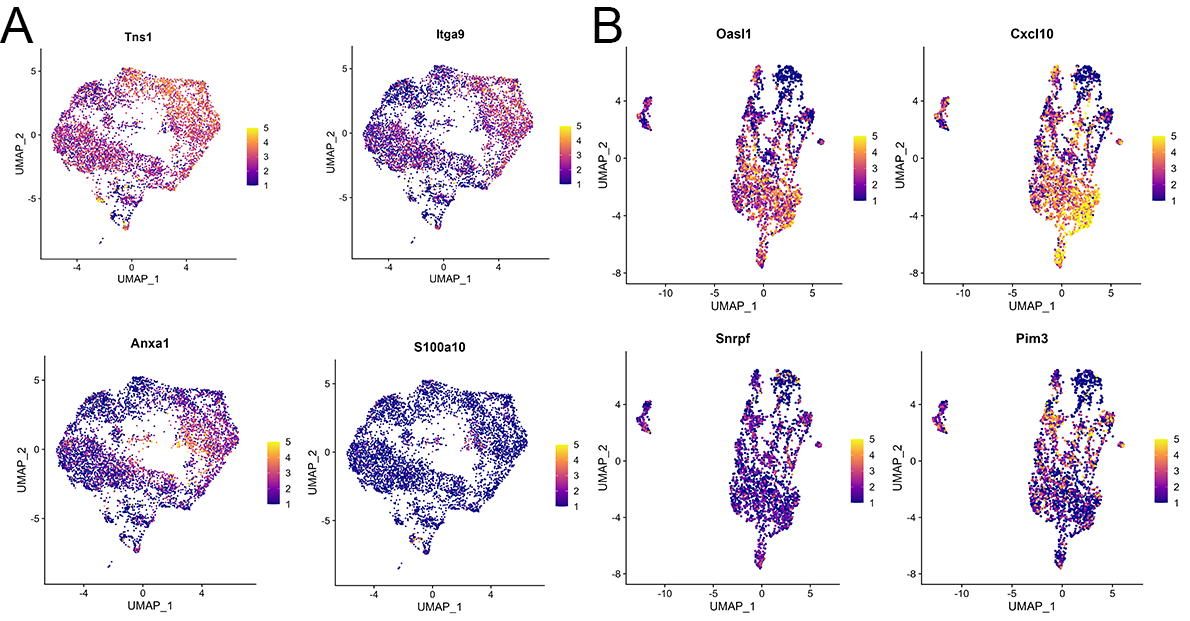


Figure S3. **Differentially expressed genes in secretory VSMCs and M1-like macrophages.** A. UMAP plot showing the expression of differentially expressed genes (Tns1, Itga9, Anxa1, and S100a10) in secretory VSMCs. B. UMAP plot showing the expression of differentially expressed genes (Oasl1,


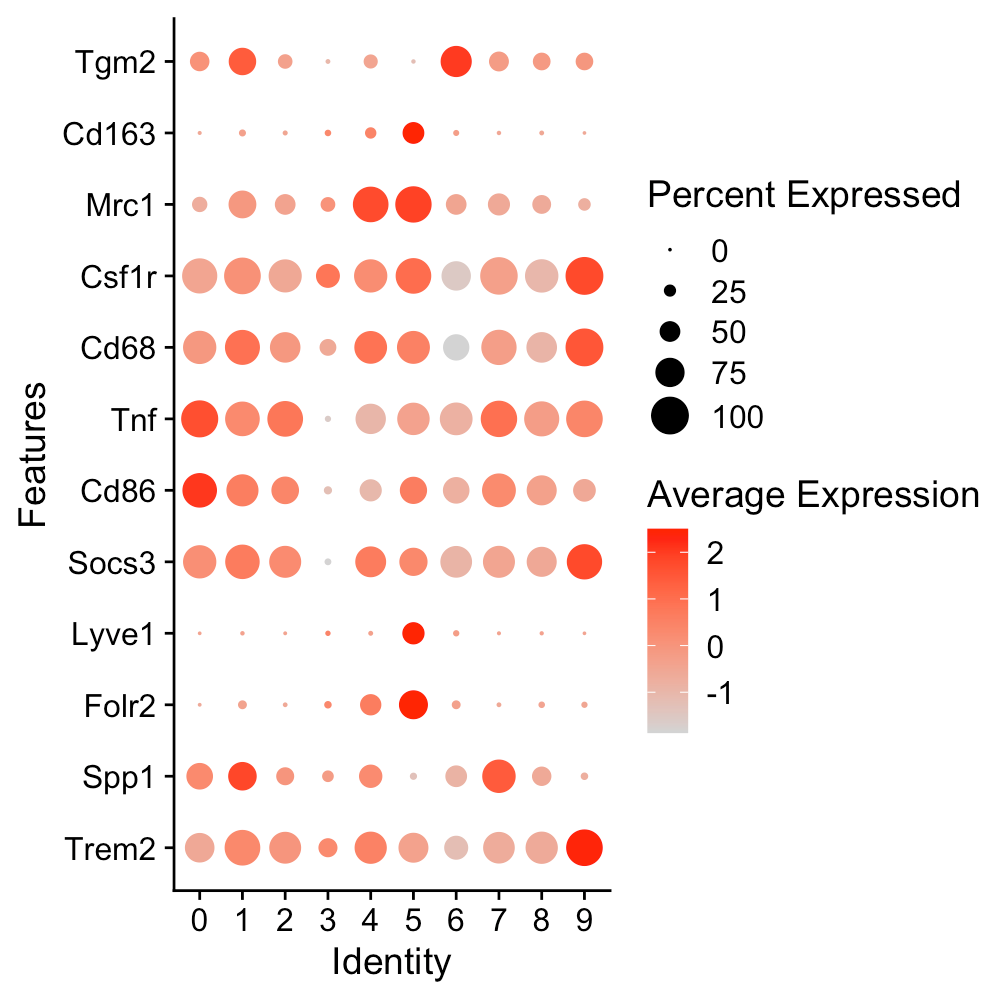


Figure S4. **Marker expression of macrophages under different classification systems.** The dotplot shows that various markers of macrophages fail to meet the exclusivity requirements of the novel classification (dichotomous classification of TREM2+SPP1+ and FOLR2+ macrophages).


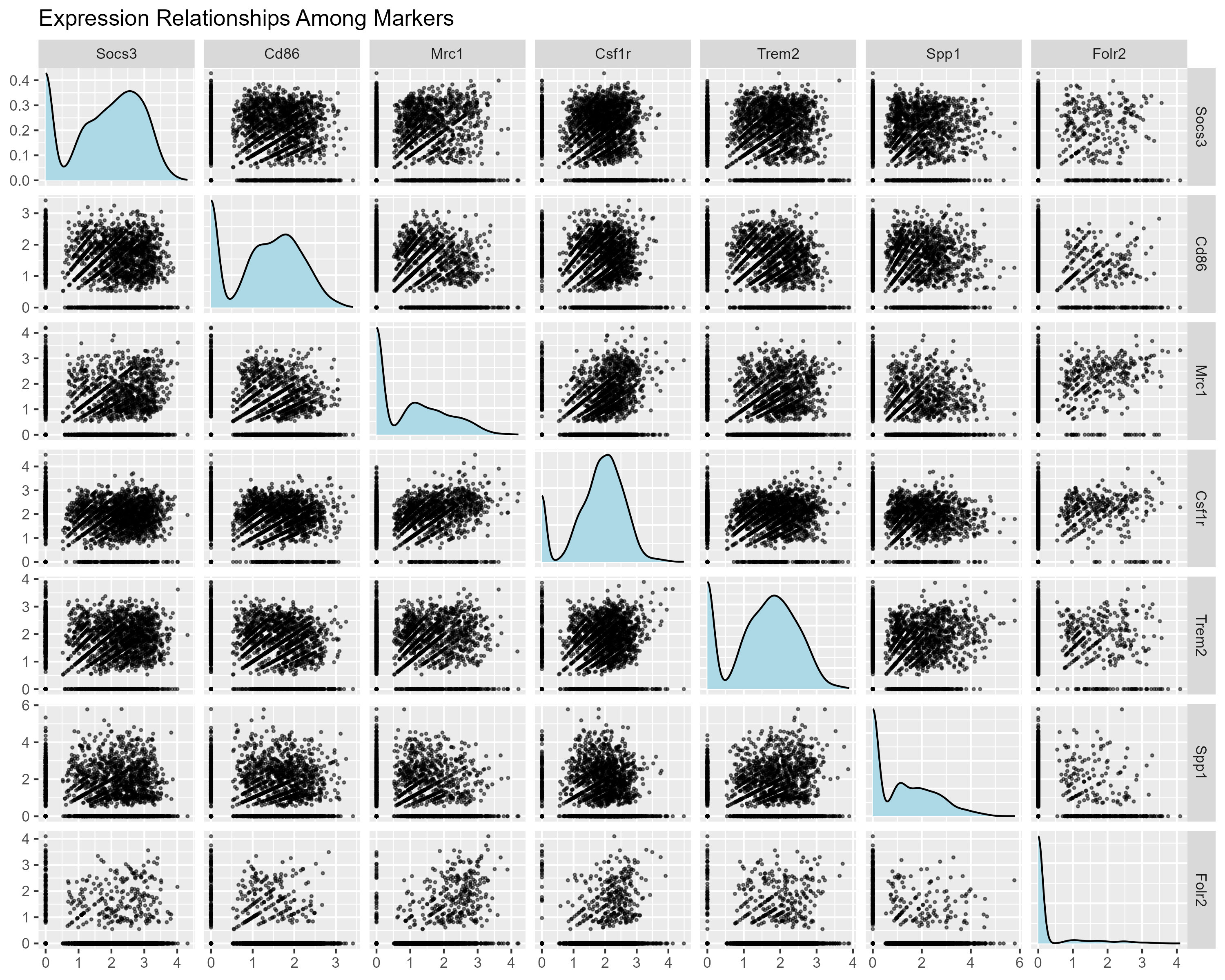


**Figure S5: Validation of Expression Exclusivity Between Classic M1/M2 Core Markers.**

Diagonal panels show kernel density estimates of Cd86 and Mrc1 expression distributions at varying levels, while off-diagonal scatterplots depict their expression correlation. Results demonstrate significant expression exclusivity between Cd86 (M1 marker) and Mrc1 (M2 marker) in high-dimensional data, with this mutually exclusive pattern consistently observed across multi-omics dimensions.


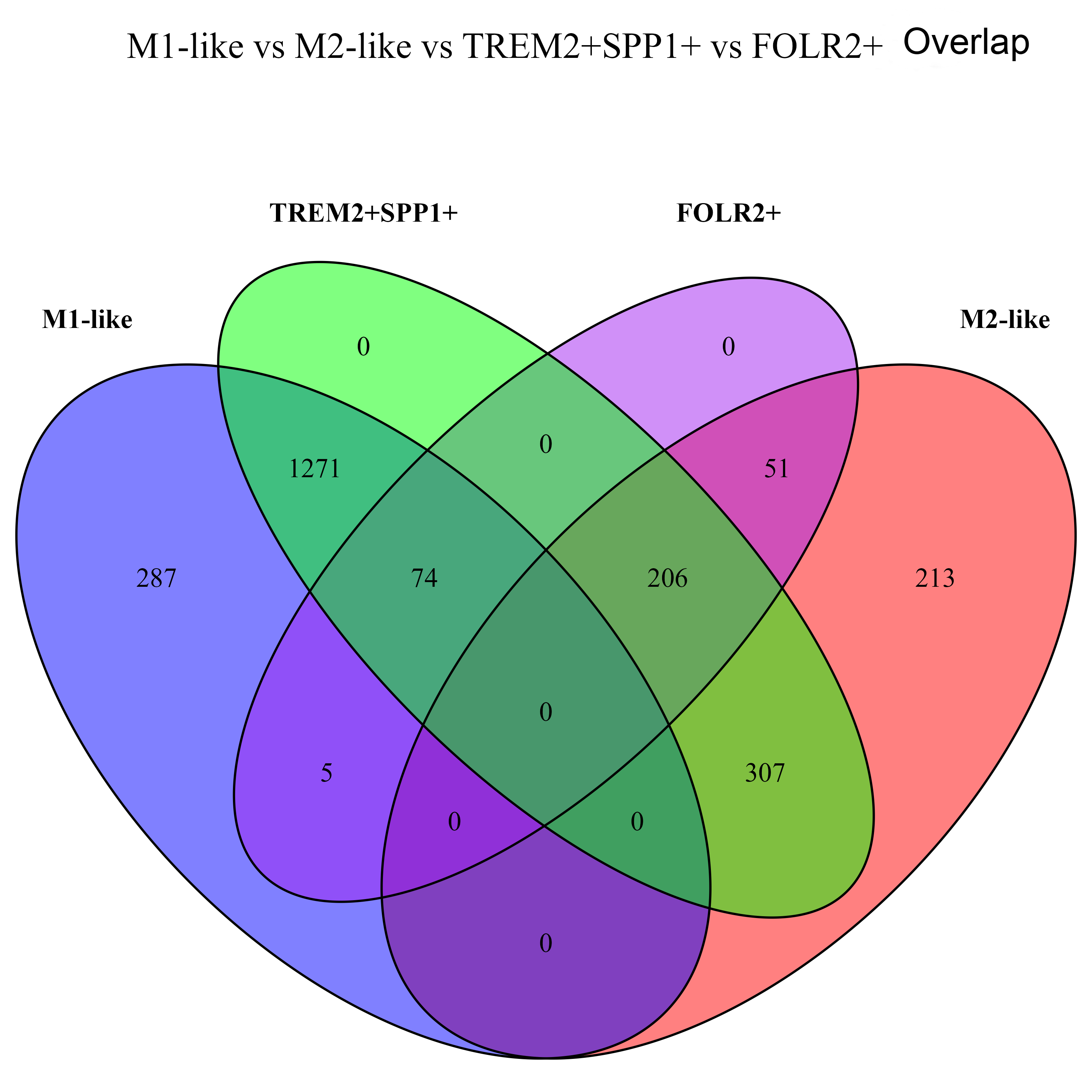


**Figure S6. Analysis of Marker Overlap Among Four Macrophage Subpopulations**.No shared markers were detected between M1-like and M2-like subgroups (intersection = 0), nor between these and TREM2+SPP1+ or FOLR2+ subgroups, indicating stable mutual exclusivity of classic M1 and M2 core markers in high-dimensional data and supporting their classification validity in pathological contexts. Although significant overlap exists between TREM2+SPP1+ and FOLR2+ subpopulations, their dual or multiple intersections with other subgroups (e.g., 1271 markers shared between TREM2+SPP1+ and M1-like; 51 markers between FOLR2+ and M2-like) imply a lack of marker exclusivity, failing to meet the critical criterion of mutual exclusivity for definitive classification.

Cxcl10, Snrpf, and Pim3) in M1-like macrophages.


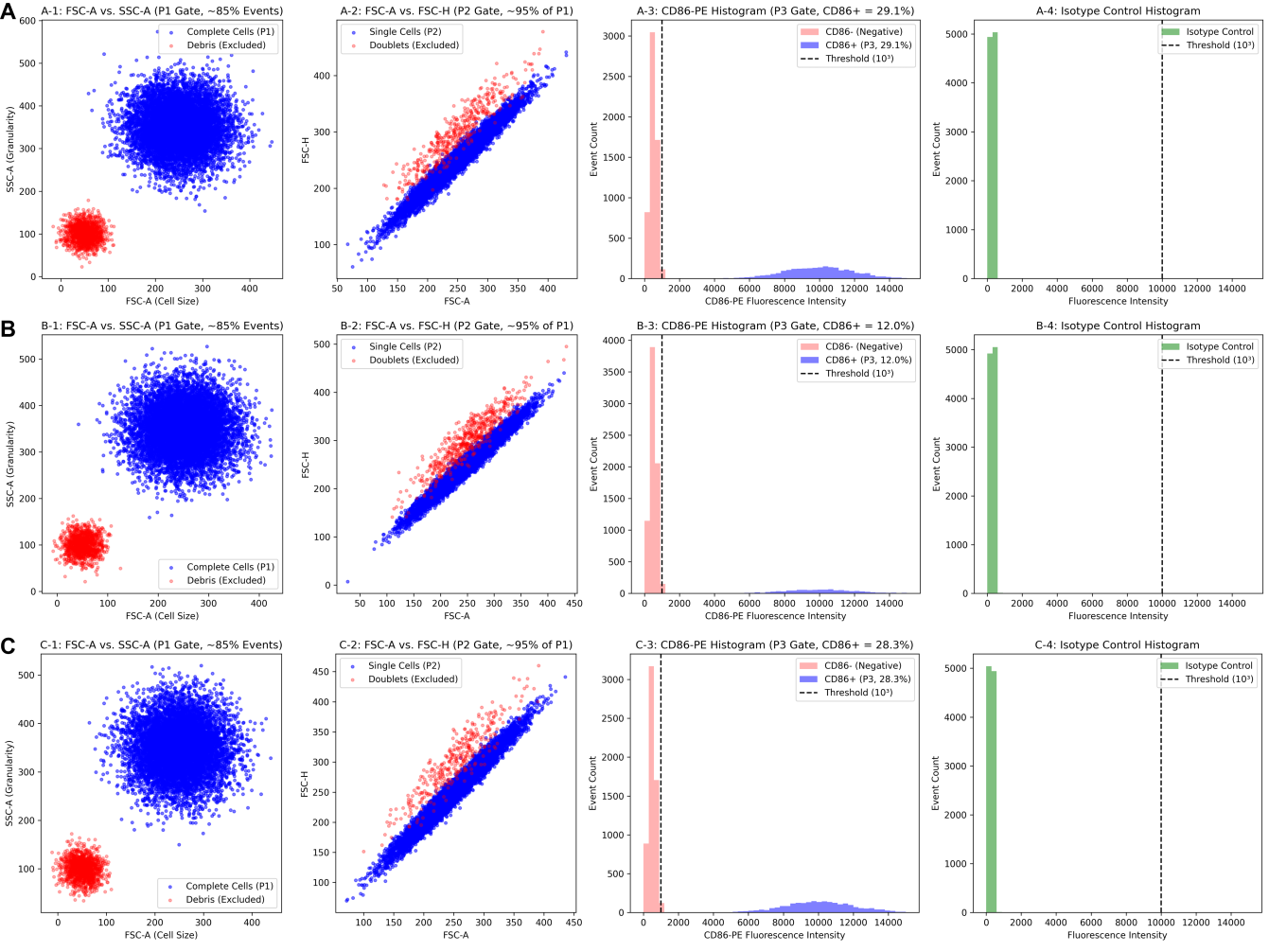
Figure S7. Flow Cytometry Gating Strategy and CD86+ Macrophage Quantification.

(A) Transwell Co-culture / Sec-VSMC Group:

A-1 (FSC-A/SSC-A): Live cells gated (P1, blue) excluding debris (red), ~85% of total events (n=10,000 cells/sample).

A-2 (FSC-A/FSC-H): Single cells gated (P2, blue), excluding doublets (red), 95% of P1.

A-3 (CD86-PE histogram): CD86+ cells (fluorescence >10,000, blue peak) constitute 29.1% ± 1.8% of single cells; threshold at 10,000 (black dashed line).

A-4 (Isotype control): Low nonspecific fluorescence (green histogram, peak 50–200), confirming CD86 specificity.

(B) MIF Knockdown / MIF-KD Sec-VSMC Group

B-1 (FSC-A/SSC-A): Live cells gated (P1), 88%.

B-2 (FSC-A/FSC-H): Single cells (P2), 94% of P1.

B-3 (CD86-PE histogram): CD86+ cells at 12.0% ± 1.2%, significantly reduced vs. A-3 (P<0.01); threshold 10,000.

B-4 (Isotype control): Similar low background fluorescence as A-4.

(C) Conditioned Medium / Sec-VSMC-CM Group

C-1 (FSC-A/SSC-A): Live cells (P1), 86%.

C-2 (FSC-A/FSC-H): Single cells (P2), 96% of P1.

C-3 (CD86-PE histogram): CD86+ cells 28.3% ± 1.5%, comparable to A-3 and significantly higher than B-3 (P<0.01).

C-4 (Isotype control): Low background fluorescence, confirming CD86 signal specificity.
