# Supplementary material for: Macrophage migration inhibitory factor–CD74 axis drives vascular smooth muscle cell–induced M1 macrophage polarization to exacerbate intracranial aneurysm inflammation
Source: Front Immunol. 2025 Nov 26;16:1682762. doi: 10.3389/fimmu.2025.1682762 (PMC12689327; doi:10.3389/fimmu.2025.1682762)
Supplement: Supplementary file 2 [file Supplementaryfile1.pdf]

frontiers

The International Society for the Advancement of Cytometry (ISAC) has highlighted the importance of including comprehensive methodological information to ensure data reproducibility and reliability. In line with this, Frontiers in Immunology now requires authors to submit a checklist for manuscripts that involve flow or mass cytometry. This checklist helps standardize the reporting process, improving the quality and transparency of published data. By doing so, we support scientific progress, making it easier for other researchers to replicate and validate experiments.

This form should be submitted with any manuscripts using flow or mass cytometry.

Sample/specimen/material description

☐ Total blood

☐ PBMCs

☐ Organ digests

Other Human VSMCs and THP-1 macrophages

Did the samples suffer any treatment before or after incubation with the antibodies?

☐ Drug ____________________________________

☑ Cell permeabilization ______________________

☐ Dye _____________________________________

☐ Propidium iodine

☐ Not applicable

Other MIF knockdown, CD74 inhibitor treatment (BMS-582949).

Instrument and antibodies

Name of the Cytometer Beckman Coulter CytoFLEX

**Antibodies and targets**	**Fluorochrome/ Metal**	**Catalog number/Company**
~~e.g. anti-CD4~~	FITC	Cat. XXX/ XXX ltd.
Anti-CD86-PE antibody	Anti-CD86-PE antibody	BioLegend, Catalog Number: 305604

Anti-human MIF monoclonal antibody	-	Abcam,ab109572
Anti-CD74 inhibitor	-	MedChemExpress,HY-12811

Data analyses

1. Name of the software R software (version 4.3.3)________
2. Reference gating strategy in the manuscript or supplementary material

Gating strategy in (eg Figure X) Gating strategy in (Figure S7).__
